# Differences in Urinary Arsenic Metabolites between Diabetic and Non-Diabetic Subjects in Bangladesh

**DOI:** 10.3390/ijerph10031006

**Published:** 2013-03-12

**Authors:** Saika Nizam, Masashi Kato, Hiroshi Yatsuya, Md. Khalequzzaman, Shoko Ohnuma, Hisao Naito, Tamie Nakajima

**Affiliations:** 1 Department of Occupational and Environmental Health, Nagoya University Graduate School of Medicine, 65 Tsurumai-cho, Showa-ku, Nagoya 466-8550, Japan; E-Mails: saikanizam@yahoo.com (S.N.); romenraihan@yahoo.com (M.K.); naitoh@med.nagoya-u.ac.jp (H.N.); 2 Department of Biomedical Sciences, College of Life and Health Sciences, Chubu University, 1200 Matsumoto-cho, Kasugai-shi, Aichi 487-8501, Japan; E-Mails: katomasa@isc.chubu.ac.jp (M.K.); shoko-oonm@aichi.email.ne.jp (S.O.); 3 Department of Public Health, Fujita Health University School of Medicine, 1-98 Dengakugakubo, Kutsukake-cho, Toyoake-shi, Aichi 470-1192, Japan; E-Mail: h828@med.nagoya-u.ac.jp

**Keywords:** arsenic, metabolites, diabetes, Bangladesh, urine

## Abstract

Ingestion of inorganic arsenic (iAs) is considered to be related to the development of diabetes mellitus. In order to clarify the possible differences in the metabolism in diabetics, we measured urinary iAs metabolites in diabetic cases and non-diabetic control subjects in Faridpur, an arsenic-contaminated area in Bangladesh. Physician-diagnosed type 2 diabetic cases (140 persons) and non-diabetic controls (180 persons) were recruited. Drinking water and spot urine samples were collected. Mean concentrations of total arsenic in drinking water did not differ between cases (85.1 μg/L) and controls (85.8 μg/L). The percentage of urinary iAs (iAs%) was significantly lower in cases (8.6%) than in controls (10.4%), while that of dimethylarsinic acid (DMA%) was higher in cases (82.6%) than in controls (79.9%). This may have been due to the higher secondary methylation index (SMI) in the former (11.6) rather than the latter (10.0). Adjusting for matching factors (sex and unions), and the additional other covariates (age and water arsenic) significantly attenuated the differences in iAs%, SMI, and DMA%, respectively, though the difference in monomethylarsonic acid% was newly significant in the latter adjustment. Our study did not suggest any significant differences in urinary arsenic metabolites between diabetic and non-diabetic subjects.

## 1. Introduction

Globally, over 100 million people are exposed to arsenic from drinking contaminated groundwater [1,2]. Arsenic contamination of the groundwater in Bangladesh is reported to be the World’s greatest calamity in terms of the population affected, since more than 50 million people in Bangladesh are chronically exposed to inorganic arsenic (iAs) concentrations exceeding the World Health Organization’s allowable standard (10 µg/L) [3,4].

Inorganic arsenate (As^V^) or arsenite (As^III^) are the usual forms of arsenic found in drinking water [5,6]. Reduction and oxidative methylation are the two major processes that occur in the metabolism of iAs in the body [7,8,9,10,11,12]. As^V^ is reduced to As^III^, which is then methylated to monomethylarsonic acid (MMA) and dimethylarsinic acid (DMA) consecutively, both of which can be rapidly excreted into urine [13]. The standard profile of urinary arsenic in humans is as follows: 10–30% of total urinary arsenic is iAs; 10–20%, MMA; and 60–80%, DMA [14]. The ratios of MMA to iAs and DMA to MMA in urine are considered indicators of the iAs metabolism, and are called the primary methylation index (PMI) and secondary methylation index (SMI), respectively [15,16,17]. Although the methylation of iAs is considered to be a detoxification process, the reduction of As^V^ to As^III^ in the methylation pathway can be viewed as a mechanism for activation of inorganic arsenic as a toxin and a carcinogen [8,18]. The disease risks associated with inter-individual variability in iAs methylation have not yet been fully understood, especially with regard to whether specific methylated arsenic species increase or decrease disease susceptibility. To date, a higher percentage of MMA (*i.e.*, a lower SMI) has been associated with malignant and pre-malignant skin lesions, bladder cancer, lung cancer, black foot disease, and peripheral vascular disease [19,20,21,22,23,24]. Tseng *et al.* [25], in their prospective follow-up study, observed that ingested iAs is diabetogenic in human beings. Study conducted in Bangladesh did not support an association of arsenic exposure from drinking water and a significantly increased risk of diabetes mellitus [26]. However, the question of whether diabetes cases have an altered iAs metabolism as represented by their urinary metabolites has yet to be determined. If found to have a different iAs metabolism, diabetics in arsenic-contaminated areas might require additional monitoring for other diseases. This hypothesis would be relevant since exposure to arsenic is known to increase the risk of developing diabetes [27,28,29]. Thus, we conducted a case-control study to investigate the possible difference in iAs metabolites in urine between diabetic cases and non-diabetic controls.

## 2. Materials and Methods

### 2.1. Study Population

The study was conducted in five unions of the Faridpur District, which is situated 130 km southwest of Dhaka, where the groundwater is known to be contaminated with arsenic. The number of inhabitants in the five unions aged 20 years or above was 71,353 [30]. We recruited cases and controls through a series of community meetings attended by 1,210 potential participants. In brief, participants were required to be aged 20 years or older, to reside in one of those five unions, and to have regularly drunk water from the same tube well for 1 year or more. Pregnant women were excluded. Community meetings were held by community leaders called “Union Chairman” for the sake of this research. The Union Chairman of each union invited individuals with diabetes and other individuals. In each union we first identified physician-diagnosed diabetic cases (of less than 5 years) through their self-reports, which were confirmed by their diabetes record books provided by the Bangladesh Institute of Research and Rehabilitation for Diabetes, Endocrine and Metabolic Disorders (Faridpur, Bangladesh) at the time of the diagnosis. All of them had a HbA1c level >7% at the time of diagnosis. Most of the included diabetes cases were thought to be on medication, but unfortunately, the majority of them revealed higher blood sugar levels and were not as well-controlled as in the other settings [31]. In total, we recruited 140 diabetic cases. Then, we selected one or more non-diabetic controls from individuals of the same sex as the index case in each union. Due to unavailability of some urine samples, the final number of male controls was less than that of male cases. Of the recruited controls, six persons were found to have a random plasma glucose level 200 mg/dL or more at the time [32], therefore, they were excluded. We also ascertained that the recruits did not have any symptoms characteristic of diabetes, such as polyuria, polydipsia, and unexpected weight loss, and chronic diseases. Cases and controls were sampled from July to August 2010. Finally, we enrolled 180 controls. Both cases and controls provided their written informed consent. The study was approved by the Ethics Review Committee of the Nagoya University School of Medicine.

### 2.2. Measurements

An interviewer-administered questionnaire was used to collect information on each respondent’s age, history of drinking water, family history of diabetes, monthly income, and smoking habit or betel nut chewing as potential confounding variables [33]. The standing height and weight of each participant were measured, with the subjects wearing light clothing and no shoes, following the standard procedures. Height was measured in centimeters using a locally made wooden measuring scale. Weight was measured in kilograms using a standard scale, which was calibrated daily and returned to zero before each measurement.

### 2.3. Urine Sample Collection and Analysis

At the time of recruitment, a spot urine sample was obtained from each participant in a paper cup, poured into a 15 mL polypropylene container, and immediately placed in a portable cooler. It was stored at −20 °C in a refrigerator without any additive. Samples were then shipped in dry ice to Dhaka, repackaged with more dry ice, shipped to the Department of Occupational and Environmental Health, Nagoya University, Japan, and stored at −80 °C in a refrigerator. We analyzed the urine samples at the Department of Biomedical Health Sciences at Chubu University, Japan. The defrosted samples were diluted five-fold with ultrapure water (specific electrical resistance 18.2 MΩ), and filtered through a 0.45 µm polytetrafluoroethylene membrane filter (Millex-LH 25 mm syringe filter; Millipore Ireland Ltd., Carrigtwohill, Ireland) to remove the turbidity. 

For the separation of arsenic compounds, we used anion-exchange column modes. In this procedure, a Shimadzu high-performance liquid chromatograph (HPLC, SCL-10 AVP, LC- 20 AD, CTO-20 AC, Shimadzu Corporation, Kyoto, Japan) and an Agilent ICP-MS (7500 cx, Agilent Technologies Inc., Palo Alto, CA, USA) were used for separating arsenic species and detecting arsenic, respectively. The instrument conditions we used for ICP-MS were as follows: RF power, 1,600 W; a plasma argon gas flow rate of 15 L/min; a carrier argon gas flow rate of 0.9 L/min; a make-up argon gas flow rate of 0.2 L/min; a micromist nebulizer, a nickel sampling cone, and a skimmer cone. We used an anionic column (Excelpak G 1836A Opt 101; and a guard column G 1836A Opt 102, Agilent Technologies, Palo Alto, CA, USA) under the following conditions: mobile phase 2.0 mM PBS/0.2 mMEDTA at pH 6.0 (adjusted with sodium hydroxide solution); flow rate, 1.0 mL/min; column temperature, room temperature; and injection volume, 20 µL. Though we measured As^III^ and As^V^ separately, we expressed total iAs as the sum of As^III^ and As^V^, since As^III^ is easily oxidized to As^V^ during sample transport, storage and preparation. Arsenobetaine (AsB) although measured, was undetected in our measurement. The limits of detection for As^III^, As^V^, MMA, DMA, and AsB were 0.1, 0.3, 0.2, 0.2 and 0.1 µg/L, respectively. Coefficients of variance (CV) for total iAs (As^V^+As^III^), MMA and DMA in day-to-day were 3.4%, 4.2% and 2.7%, respectively, and in the day 2.8%, 1.3% and 2.2%, respectively, when we used 50 ppb standard solution.

### 2.4. Water Sample Collection and Analysis

Drinking water samples were collected from 195 tube wells identified by each participant as his or her primary source of drinking water. The number of tube wells was smaller than that of total participants (n = 320) since some tube wells was shared by multiple participants. The tube wells were pumped for several minutes for purging before 15 mL of water was collected in polypropylene containers. Samples were preserved without any additive at −20 °C, transferred to Dhaka, shipped to the Department of Occupational and Environmental Health, Nagoya University, Japan, and kept at −80 °C in a refrigerator until analysis. As with the urine, water samples were analyzed in the Department of Biomedical Sciences at Chubu University, Kasugai, Japan, using an inductively coupled plasma-mass spectrometer (ICP-MS; 7500cx, Agilent Technologies Inc., Palo Alto, CA, USA) which has a detection limit of 0.1 µg/L. 

### 2.5. Data Analyses and Statistical Methods

Total arsenic (TAs) was defined as the sum of iAs, MMA, and DMA. In our analytical conditions with ICP-MS, we could not differentiate between trivalent and pentavalent forms of arsenic. The proportions of urinary iAs and its metabolites were expressed as iAs/TAs (iAs%), MMA/TAs (MMA%), and DMA/TAs (DMA%). To assess the arsenic methylation capacity, two methylation indices, the primary methylation index (PMI) and the secondary methylation index (SMI), were calculated as MMA/iAs and DMA/MMA, respectively.

Differences between cases and controls were compared by three-way analysis of covariance with matching factors (sex and union) as the fixed factors. Variables with a skewed distribution were logarithmically transformed before statistical tests. Multivariate-adjusted means were estimated by general linear models with the same fixed factor crude value (Model 1) and covariates, including log-transformed water arsenic and age (Model 2), variables in Model 2, smoking habit, family history of diabetes, years of using the tube well for drinking water, and body mass index (BMI) (Model 3). BMI was calculated as the measured weight in kilograms divided by the measured height in meters squared. BMI and monthly income were categorized by 25 kg/m^2^ and median (147 USD), respectively. All the calculations were performed using the Statistical Package for the Social Sciences (SPSS) for windows, version 20 software (IBM Japan, Tokyo, Japan). A two-tailed *P* < 0.05 was considered to indicate statistical significance.

## 3. Results

Diabetic cases were older than controls (48.0 *vs.* 41.4 years, *P* ≤ 0.001, Table 1), and they had a significantly longer family history of diabetes than the controls (46.4% *vs.* 22.2%, *P* ≤ 0.001). Monthly incomes were significantly higher in cases (230 USD) than controls (165 USD) (*P* ≤ 0.001). We found no significant difference in the duration of using the tube well for drinking water or in the arsenic concentration of the well water used between cases and controls. Random plasma glucose concentrations were significantly higher in diabetic cases than in the controls (*P* = 0.001). No significant differences were observed in the prevalence of habitual smoking or betel nut chewing, or in the mean BMI between cases and controls. There were also no differences in absolute urinary arsenic profiles of TAs, iAs, MMA and DMA between cases and controls.

**Table 1:** Means and percentages of diabetic cases and controls, Faridpur, Bangladesh.

Variables	Diabetic cases (n = 140)	Controls (n = 180)	* *P*-value
Demographic			
Sex			0.037
Male	70 (50)	69 (38)	
Female	70 (50)	111 (62)	
Age (years)	48.0 (46.3–49.7)	41.4 (39.7–43.2)	≤0.001
Family history of diabetes			≤0.001
Yes	65 (46.4)	40 (22.2)	
No	75 (53.6)	140 (77.8)	
Monthly income (USD)	230 ± 186	165 ± 86	≤0.001
Smoking habit			0.438
Yes	23 (16.4))	24 (13.3)	
No	117 (83.6)	156 (86.7)	
Betel nut chewing			0.928
Yes	45 (32.1)	57 (31.7)	
No	95 (67.9)	123 (68.3)	
No education	13 (9)	30 (17)	
Primary	61 (44)	82 (46)	
Secondary and above	66 (47)	68 (37)	
	Water		
Arsenic in drinking water (μg/L)	85.1 (69.3–100.9)	85.8 (74.7–96.9)	0.946
Water arsenic level (μg/L)			0.241
<10	24 (17)	23 (13)	
10–50	48 (34)	53 (29)	
>50	68 (49)	104 (58)	
Duration of water drinking (years)	11.7 (9.8–13.6)	11.8 (10.4–13.3)	0.926
	Test		
BMI (kg/m^2^)	25.1 (25.5–25.6)	24.5 (23.8–25.1)	0.150
BMI (kg/m^2^)			0.070
≤25	62 (44)	98 (54)	
>25	78 (56)	82 (46)	
Random plasma glucose (mg/dL)	253 (237–270)	125 (121–128)	0.001
Urinary creatinine (mg/dL)	183.0 (164–202)	175.0 (160–190)	0.500
Absolute values (μg/L)			
TAs	252.2 (208.3–296.0)	235.3 (197.6–273.0)	0.565
iAs	20.0 (15.7–23.8)	21.2 (17.9–24.5)	0.650
MMA	23.9 (18.7–29.0)	22.2 (18.5–25.7)	0.595
DMA	208.3 (172.7–243.9)	192.0 (159.9–224.0)	0.813

Values in Table indicate n (%) or mean (95% confidence interval). Monthly income is expressed as mean ± standard deviation. TAs, Sum of (iAs + MMA + DMA). iAs, Inorganic arsenic. MMA, monomethylarsonic acid. DMA, dimethylarsinic acid. * By chi-squared test or Student *t*-test. *USD*, United States Dollar. *BMI*, Body mass index.

Table 2 shows a comparison of urinary arsenic profiles among subgroups of age, sex, smoking habit, family history of diabetes, BMI, monthly income, water arsenic level in the wells and the duration of drinking the water. Levels of iAs% and MMA% were significantly higher in males than in females, while DMA% and SMI were significantly higher in females. No sex differences were observed in absolute values of iAs, MMA and PMI. Age significantly influenced iAs% and PMI, but not other arsenic profiles. There was no difference in arsenic profiles between subjects with and without a family history of diabetes and also in the smoking habit. Subjects with high incomes had lower iAs%, but higher DMA%. However, there were no differences in other arsenic profiles between them. Subjects whose well water contained arsenic >50 µg/L had significantly higher absolute iAs, MMA, DMA, TAs, and MMA% compared to those using less contaminated well water. Interestingly, the arsenic concentration of the well water did not influence iAs% and DMA% or PMI and SMI, and the duration of drinking the well water did not influence arsenic profiles. 

**Table 2:** Comparison of urinary arsenic profiles in absolute values and percentages between subgroups, Faridpur, Bangladesh, 2010.

Variables	n	iAs μg/L (%)	MMA μg/L (%)	DMA μg/L (%)	TAs μg/L	PMI	SMI
Sex							
Male	139	20.2 (10.5)	23.3 (10.3)	183.3 (79.1)	226.8	1.2	9.2
Female	181	21.0 (8.9)	22.6 (8.7)	212.4 (82.4)	255.0	1.3	11.7
*P-*value		NS (0.020)	NS (0.001)	NS (0.001)	NS	NS	0.001
Age (years)							
20–44	157	21.7 (10.5)	21.1 (9.3)	185.7 (80.2)	228.6	1.1	10.8
45–70	163	19.2 (8.8)	23.8 (9.6)	208.3 (81.6)	251.4	1.4	10.4
*P-*value		NS (0.016)	NS (NS)	NS (NS)	NS	0.005	NS
Family history of diabetes							
Yes	105	19.3 (9.9)	22.7 (9.5)	199.2 (80.7)	241.2	1.1	10.7
No	215	22.3 (10.1)	23.5 (9.7)	198.5 (80.1)	244.1	1.2	10.1
*P-*value		NS (NS)	NS (NS)	NS (NS)	NS	NS	NS
Monthly income (United States Dollars)							
Low group	165	23.2 (10.4)	24.6 (9.8)	203.7 (79.8)	251.6	1.2	10
High group	155	18.3 (8.7)	21.3 (9.0)	193.0 (82.1)	232.6	1.3	10.9
*P-*value		NS (0.025)	NS (NS)	NS (0.018)	NS	NS	NS
Smoking habit							
Yes	47	18.1 (9.8)	20.5 (10.3)	147.0 (80.0)	185.7	1.1	9.2
No	273	21.0 (9.6)	23.4 (9.3)	209.1 (81.1)	253.4	1.3	10.8
*P-*value		NS (NS)	NS (NS)	NS (NS)	NS	NS	NS
Water arsenic (μg/L) ^a^							
≤50	148	14.4 (9.5)	15.3 (8.9)	149.0 (81.6)	178.7	1.3	11.3
>50	172	26.1 (9.8)	30.0 (9.9)	245.5 (80.3)	301.5	1.3	9.9
*P-*value		0.001 (NS)	0.001 (0.020)	0.001 (NS)	0.001	NS	NS
Duration of drinking water (years) ^b^							
≤10	159	21.8 (10.1)	23.5 (9.7)	204.0 (80.2)	249.3	1.2	11.3
>10	156	19.8 (9.1)	23.0 (9.1)	200.7 (81.7)	243.5	1.3	10.8
*P-*value		NS (NS)	NS (NS)	NS (NS)	NS	NS	NS
BMI (kg/m^2^)							
≤25	160	22.2 (9.9)	23.9 (10.0)	197.6 (80.0)	243.7	1.3	10.7
>25	160	19.1 (9.3)	22.1 (8.8)	203.1 (81.9)	244.2	1.3	11.3
*P-*value		0.050 (NS)	NS (0.030)	NS (0.046)	NS	NS	0.02

^a^ The number of tube wells was 195 instead of 320, since some tube wells were shared by multiple participants; ^b^ Five respondents failed to recall the duration of drinking water; NS indicates not significant (*P* > 0.05); BMI indicates body mass index; iAs indicates inorganic arsenic, and iAs% in parentheses is calculated as ((arsenite + arsenate)/total urinary arsenic) × 100; MMA indicates monomethylarsonic acid, and MMA% in parentheses is calculated as (MMA/total urinary arsenic) × 100; DMA indicates dimethylarsinic acid and DMA% in parentheses is calculated as (DMA/total urinary arsenic) × 100; TAs is the sum of (iAs + MMA + DMA); PMI indicates primary methylation index calculated as MMA/iAs; SMI indicates secondary methylation index calculated as DMA/MMA.

Overweight participants (BMI > 25 kg/m^2^) had significantly lower absolute values of iAs, but significantly higher DMA% and SMI than normal weight individuals. Thus, only differences in PMI were observed with age, while differences in PMI and SMI were observed, both in gender and BMI. Crude and adjusted means of the percentage of urinary iAs and its metabolites in diabetic cases and controls are presented in Table 3. 

**Table 3:** Adjusted means of urinary inorganic arsenic, its metabolites and methylationindexes in cases and controls.

Characteristics	Diabetic cases (n = 140)	Controls (n = 180)	* *P*-value
iAs%			
^a^ Crude	8.6	10.4	0.022
^b^ Model 1	9.0	10.8	0.065
^c^ Model 2	9.3	10.4	0.281
^d^ Model 3	9.5	10.5	0.345
MMA%			
^a^ Crude	8.8	9.7	0.064
^b^ Model 1	9.4	10.4	0.068
^c^ Model 2	9.3	10.5	0.041
^d^ Model 3	9.3	10.3	0.081
DMA%			
^a^ Crude	82.6	79.9	0.012
^b^ Model 1	81.6	78.8	0.028
^c^ Model 2	81.3	79.1	0.087
^d^ Model 3	81.2	79.3	0.142
PMI			
^a^ Crude	1.4	1.2	0.191
^b^ Model 1	1.3	1.2	0.488
^c^ Model 2	1.3	1.3	0.641
^d^ Model 3	1.2	1.3	0.365
SMI			
^a^ Crude	11.6	10.0	0.037
^b^ Model 1	10.7	9.2	0.089
^c^ Model 2	10.9	9.1	0.053
^d^ Model 3	10.8	9.3	0.093

^a^ Crude value; ^b^ Model 1 adjusted for matching factors (sex and union); ^c^ Model 2 adjusted for variables in Model 1, age and water arsenic; ^d^ Model 3 adjusted for variables in Model 2, monthly income, duration of drinking water, family history of diabetes, smoking habit and body mass index; * By three-way analysis of covariance with case-control and matching factors (sex and union) as the fixed factors.

The crude values of iAs% were significantly lower in cases than controls (*P* = 0.022). In contrast, those of DMA% and SMI were significantly higher in diabetic cases. The significant differences in iAs% and SMI were attenuated by using matching factors (sex and union) in Model 1, while the difference in DMA% was retained. After further adjustments for age and water arsenic (Model 2), differences observed in crude and Model 1 values disappeared, while a significant difference in MMA% was newly observed between cases and controls (Model 2). It should be noted that in Model 2, the difference in SMI was borderline between cases and controls. Interestingly, further adjustments of Model 2 for income, duration of using the tube well for drinking water, family history of diabetes, smoking habit, and BMI did not retain differences in all arsenic profiles.

## 4. Discussion

In this case-control study in Bangladesh, we investigated urinary arsenic metabolites among diabetic and non-diabetic subjects. The iAs% was significantly higher in non-diabetic controls than in diabetic cases, whereas DMA% and SMI were significantly higher in cases than in controls. Although adjustment for potential confounding variables attenuated statistical significance of the differences, the differences in the percentage mean values did not alter much among the two groups after the adjustment, especially regarding MMA% and SMI. Therefore, our finding of higher MMA% and lower SMI in controls may have some physiological implications. However, since higher MMA% and lower SMI can be seen as a methylation capacity profile more prone to arsenic toxicity, the current understanding of arsenic exposure makes it difficult to consider that having this profile might have been a cause of not developing diabetes, although causality can only be inferred from a cross-sectional study. 

We attempted to explain our findings by the residual confounding of preexisting dietary and nutritional differences between diabetics and controls. Thus, even though our findings were adjusted for potential confounding due to socio-economic differences between cases and controls by monthly income, there may be residual confounding of diet and nutrition. We speculated that individuals who had consumed a “better” diet in the context of developing countries, characterized by a high caloric and high animal fat and protein diet [34,35], might have developed diabetes, and that these individuals had continued their dietary practice. In contrast, socio-economically poor individuals might have consumed less for energy expenditure and less protein, especially from animal, which could have led to their lower chance of developing diabetes. BMI or obesity is another strongest risk factor for diabetes. Since we collected prevalent diabetes cases, BMI might have been changed. Namely, they might have lost weight after the onset. Lowered arsenic methylation in people with a low protein intake has been reported [36,37,38]. Thus, higher MMA% and lower SMI in controls might have been due to their lower protein intake. Also, higher MMA% and lower SMI were observed in individuals with a BMI of 25 kg/m^2^ or lower in the present study. Since BMI could be a general indicator of nutritional status in populations where the underweight are poor [39], the methylation capacity profile observed in this study might imply their poor nutritional status. This speculation would be consistent with a previous study which showed that malnutrition had increased arsenic-induced health effects through lower antioxidant defense and alterations in the metabolism of arsenic [40]. Another study of Bangladeshi adults whose diet was deficient in folate indicated their increased susceptibility to arsenic toxicity too [41]. Folate is needed for the biochemical pathway of a one-carbon metabolism, which is essential for the methylation of arsenic metabolites. Further studies are warranted to understand and explain the present findings. 

Considering sex and smoking habit, we found that women had significantly higher DMA%, SMI and TAs, and significantly lower iAs% and MMA% than men, consistent with previous studies that found higher arsenic methylation capacity in women than men [15,23,42]. We did not find differences in the methylation capacity between smoking status in the present study, which was inconsistent with a previous study that found a deteriorated capacity associated with smoking [43]. The reason behind this may have been the small smoker populations in our study.

We detected no AsB, which would have appeared as organic arsenic in the present study. Since the study area was far from the sea and the population was not habituated to a seafood diet, TAs in the present sample most likely reflect iAs ingested mainly through drinking water. Previous studies found significantly higher levels of TAs in the urine of diabetic patients than in that of non-diabetics [44,45,46]. The higher TAs observed in diabetics in our study was not statistically significant, although it is possible that cases drank more arsenic-contaminated water due to diabetic symptoms, such as thirst.

In our study, we did not find an arsenic/metabolism difference, either from low or high arsenic exposure. Urinary arsenic metabolites have been studied in several other diseases. The importance of a complete second methylation may be implied from the observation that human beings who excrete higher MMA% in the urine are more susceptible to arsenic-induced health hazards such as lung cancer [22], skin cancer [19,20,47], and peripheral vascular disease [23]. Higher PMI and lower SMI were also related to arsenic-induced skin cancer [47], urothelial carcinoma [48], and bladder cancer [21]. In the present study, we found significantly higher MMA% in individuals whose well water contained 50 μg/L or more arsenic than their counterpart, which is consistent with a previous observation [49]. From our finding, however, it is difficult to state that arsenic detoxification is related with diabetic susceptibility, since we did not observe a significant difference in MMA% between the cases and controls.

Our findings would be consistent with Steinmaus *et al.* [50] who did not find a difference of iAs in diabetic cases and controls in a US population with a low level of exposure. Chen *et al.* [26] also did not find a difference in TAs between diabetic and non-diabetic subjects in Bangladesh. However, neither of these studies provided a detailed methylation capacity profile.

There are several limitations to the present study. First, as described earlier, we did not measure each dietary intake among the two groups. Future investigations should include valid assessment of dietary and nutritional factors. Second, although both cases and controls were selected from the same study base (community), both groups were volunteers who had attended community meetings. Future studies should also identify cases through a disease registry, in order to include patients that might have a different disease severity, or a different lifestyle from those who had actually attended. Third, we failed to match age this time as it was difficult in the setting and we would be paying serious attention in our future studies to overcome this problem. Fourth, we defined controls by the self-report of no history, absence of symptoms, and random plasma glucose level. There is a possibility that undiagnosed asymptomatic diabetics whose random glucose did not exceed 200 mg/dL were included in controls. Future studies should define healthy by HbA1c levels or create a glucose tolerance test. Fifth, we attempted to control cumulative arsenic exposure by adjusting for the water arsenic level and the duration of drinking well water. Since the drinking duration was self-reported and the well we identified was the one used most proximately, there may have been misclassification. However, it is unlikely that the diabetes status was related to the misclassification, and the difference in the cumulative arsenic exposure might have been biased toward null. Furthermore, we did not obtain the amount of rice consumption, a staple food of Bangladeshis. Since rice has been reported as an important source of arsenic exposure [51], future studies should include rice and other dietary assessments in order to appropriately control for arsenic exposure. Sixth, comorbidities such as nonalcoholic fatty liver disease, which is usually asymptomatic, can disrupt the arsenic metabolism [52]. Moreover, patients with diabetes may have altered renal function that could distort the urinary excretion of arsenic metabolites. However, due caution is required when interpreting the present findings.

## 5. Conclusions

Our study did not suggest any significant differences in urinary arsenic metabolites between diabetic and non-diabetic subjects.
